# Supplementary material for: Increased Galectin-9 Levels Correlate with Disease Activity in Patients with DMARD-Naïve Rheumatoid Arthritis and Modulate the Secretion of MCP-1 and IL-6 from Synovial Fibroblasts
Source: Cells. 2023 Jan 15;12(2):327. doi: 10.3390/cells12020327 (PMC9857341; doi:10.3390/cells12020327)
Supplement: Supplementary file 1 [file cells-12-00327-s001.zip › cells-2102291-supplementary.pdf]

Supplementary Figure S1

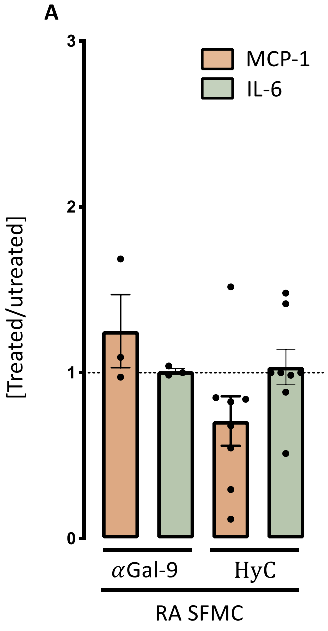

Secretion of MCP-1 and IL-6 from SFMC cultures. Results are expressed as ratios of the cytokine concentrations in neutralizing Gal-9, (Gal-Nab2, 10 μg/ml) (n = 3) and Hydrocortisone (HC) (1 μg/ml) (n = 8) treated cultures divided by the concentration in control cultures, cultured for 48 hours. Untreated RA SFMC cultures produced averagely 257.7 ±174.5 pg/ml MCP-1 and 43.23 ±9.2 pg/ml IL-6 (Mean ±SEM). Bars indicate mean +/- SEM.

Supplementary Figure S2

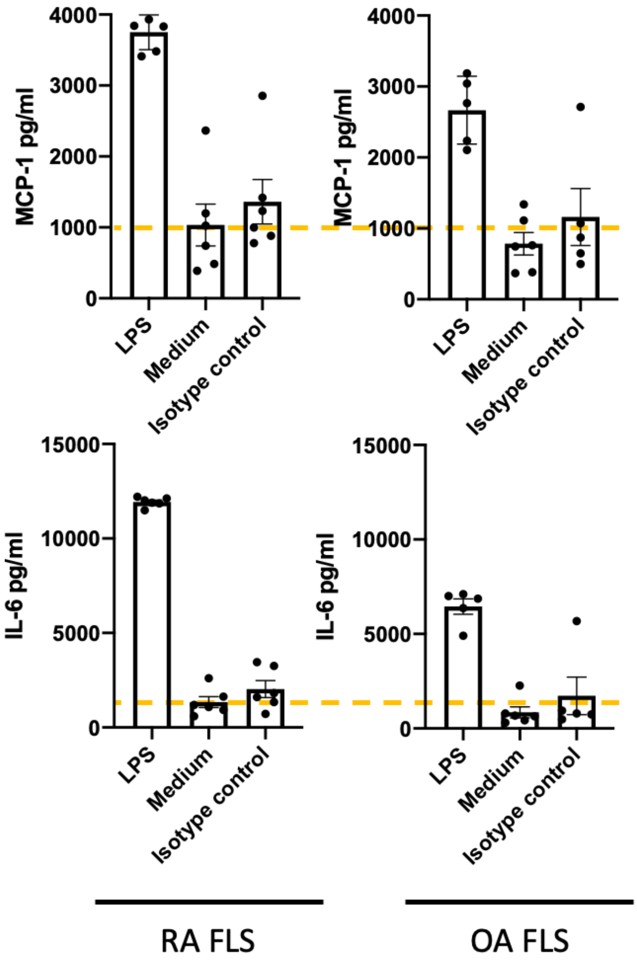

Secretion of MCP-1 and IL-6 from 48 h RA and OA FLS cultures. Bars indicate mean +/- SEM. Untreated monocultures of RA FLS (Yellow dotted line) produced averagely 1034 ±295 pg/ml MCP-1 and 1341 ±289 pg/ml IL-6 and OA FLS produced 784 ±158 pg/ml MCP-1 and 854± 293 pg/ml IL-6 (Mean ± SEM).
